# Health-seeking behaviour of human brucellosis cases in rural Tanzania

**DOI:** 10.1186/1471-2458-7-315

**Published:** 2007-11-03

**Authors:** John Kunda, Julie Fitzpatrick, Rudovic Kazwala, Nigel P French, Gabriel Shirima, Alastair MacMillan, Dominic Kambarage, Mark Bronsvoort, Sarah Cleaveland

**Affiliations:** 1 National Institute for Medical Research, Muhimbili Centre, Tanzania; 2 Centre for Tropical Veterinary Medicine, University of Edinburgh, Easter Bush, EH25 9RG, UK; 3 Moredun Research Institute, Pentlands Science Park, Midlothian, Edinburgh, EH26 0PZ, UK; 4 Sokoine University of Agriculture, Morogoro, Tanzania; 5 Institute of Veterinary, Animal and Biomedical Sciences, Massey University, New Zealand; 6 Veterinary Research Directorate, DEFRA, Cromwell House, London, SW1P 3JH, UK; 7 Animal Disease Research Institute, Dar es Salaam, Tanzania

## Abstract

**Background:**

Brucellosis is known to cause debilitating conditions if not promptly treated. In some rural areas of Tanzania however, practitioners give evidence of seeing brucellosis cases with symptoms of long duration. The purpose of this study was to establish health-seeking behaviour of human brucellosis cases in rural Tanzania and explore the most feasible ways to improve it.

**Methods:**

This was designed as a longitudinal study. Socio-demographic, clinical and laboratory data were collected from patients who reported to selected hospitals in rural northern Tanzania between June 2002 and April 2003. All patients with conditions suspicious of brucellosis on the basis of preliminary clinical examination and history were enrolled into the study as brucellosis suspects. Blood samples were taken and tested for brucellosis using the Rose-Bengal Plate Test (RBPT) and other agglutination tests available at the health facilities and the competitive ELISA (c-ELISA) test at the Veterinary Laboratory Agencies (VLA) in the UK. All suspects who tested positive with the c-ELISA test were regarded as brucellosis cases. A follow-up of 49 cases was made to collect data on health-seeking behaviour of human brucellosis cases.

**Results:**

The majority of cases 87.7% gave a history of going to hospital as the first point of care, 10.2% purchased drugs from a nearby drug shop before going to hospital and 2% went to a local traditional healer first. Brucellosis cases delayed going to hospital with a median delay time of 90 days, and with 20% of the cases presenting to hospitals more than a year after the onset of symptoms. Distance to the hospital, keeping animals and knowledge of brucellosis were significantly associated with patient delay to present to hospital.

**Conclusion:**

More efforts need to be put on improving the accessibility of health facilities to the rural poor people who succumb to most of the diseases including zoonoses. Health education on brucellosis in Tanzania should also stress the importance of early presentation to hospitals for prompt treatment.

## Background

Brucellosis is caused by gram-negative bacilli, of the genus *Brucella *(*Brucella abortus, B. suis, B. melitensis and B. canis*) [1]. The most common clinical features of brucellosis include fever, fatigue, headache, sweating, joint pain, loss of appetite, muscular pain, lumber pain, weight loss, hepatomegally, splenomegally and arthritis. The multiple and non-specific features of brucellosis contribute to difficulties in the diagnosis of brucellosis in areas where diseases with similar clinical features such as malaria, tuberculosis, typhoid and joint diseases co-exist [1,2].

Although generally speaking, any member of the public is at risk of getting brucellosis through consumption of poorly prepared dairy products in the form of meat, milk, cheese and butter, certain occupations such as veterinarians, butchers, abattoir workers, meat inspectors, farmers and those working in meat packing and dairy processing industries are known to be at a greater risk [3,4].

In many sub-Sahara African countries, febrile or flu-like conditions with similar manifestations occur commonly and have significantly contributed to difficulties in the diagnoses of such diseases as brucellosis, typhoid, malaria, amoeba and tuberculosis [5,6]. In Narok, Kenya, 12% of flu-like patients were diagnosed using the RBPT as brucellosis patients and 40% typhoid patients [7]. In Kampala, Uganda, of patients with joint pain, general malaise, and/or constant headache, 73% were found to be suffering from malaria and 13.3% from brucellosis [5].

Clinical features and laboratory investigation form the basis for the diagnosis of brucellosis in humans [8]. The definitive diagnosis of brucellosis is by culture and isolation of the causative organisms. However, the procedure requires special media, takes several weeks of incubation and has low sensitivity [9,10]. The laboratory diagnosis of brucellosis therefore often depends on serologic tests. These include the Serum Agglutination Tests (SAT) [11], the Complement Fixation Test [12], the Fluoroscent Polarization Assay [13,14] and the Enzyme-Linked immunosorbent Assays (ELISA) tests [15]. Other tests include the radioimmunoassay [16], the indirect immunofluorescence assay [17], and the 2-mercaptoethanol test (2ME) [18].

In the sub-acute or chronic phase of brucellosis however, the agglutination tests may be particularly difficult to interpret or may be negative and other tests need to be done to confirm the results. This is because the serum agglutination test depends very much on the presence of IgM immunoglobulin that could be low or absent in chronic and sub-acute states. This also explains why the SAT is negative during the incubation period and following abortion [19].

The c-ELISA test for the detection of serum antibodies to the organisms of the genus *Brucella *has been shown to be a suitable test for human brucellosis [15]. The c-ELISA test uses a monoclonal antibody (mAb) specific for a common and repeating epitope on the polysaccharide portion of the smooth lipopolysaccharide molecule of *Brucella *to compete with antibody in the sample. This results in an assay with higher specificity than other assays because it frequently eliminates cross-reactions with other antigens while retaining its high sensitivity. The c-ELISA test therefore could be adopted as the confirmatory test for human brucellosis [15].

Most of the hospital laboratories in rural sub-Saharan Africa have limited capacity for the diagnosis of brucellosis. Brucellosis is commonly diagnosed after failure to respond to malaria, typhoid or tuberculosis treatments [20,21]. In the current study, it was observed that serological diagnosis was only conducted in districts or designated district hospitals. In Kenya, local clinics were conducting the RBPT, but additional tests such as the SAT were only conducted in central veterinary or medical testing facilities [7,20]. Most of these facilities are not easily accessible to the majority of people in rural areas of Africa due to their geographical location and poor infrastructure.

Brucellosis caused by *B. melitensis *is the most important clinically apparent disease in humans and is the one usually associated with occupational exposure or consumption of poorly prepared dairy products [22], followed by infection with *B. abortus *and by *B. suis*. Because of its greater severity, infections with *B. melitensis *are generally considered more likely to be diagnosed than infection with other *Brucella *species [23].

Health-seeking behaviour of patients suffering from infectious and non-infectious diseases have been evaluated in many parts of Africa. These include those suffering from tuberculosis [24-27], sleeping sickness [28] and non-infectious diseases [29]. Factors that determine when patients get hospital treatment vary from the patients' own reasons to those due to health providers. Patient factors include expectations that the symptoms might improve, visit to local traditional healer and self medication from a nearby drug shop or private clinic.

Other factors that have been documented as causing delay in patients seeking health facilities' treatment include the distance to the nearest health facility and socio-economic status. Some households are far from hospitals and poor infrastructure make accessibility to health care difficult. In areas where there is transportation, affordability of the costs of transport made patients unable to present to hospitals in time. Factors related to health provider included poor referral system, high work load and diagnostic difficulties [24-28]. The purpose of this study was to investigate factors that play role in determining when brucellosis cases present to hospital, where they go first to seek treatment and try to come up with most feasible solutions.

## Methods

### Study area

**Figure 1:**
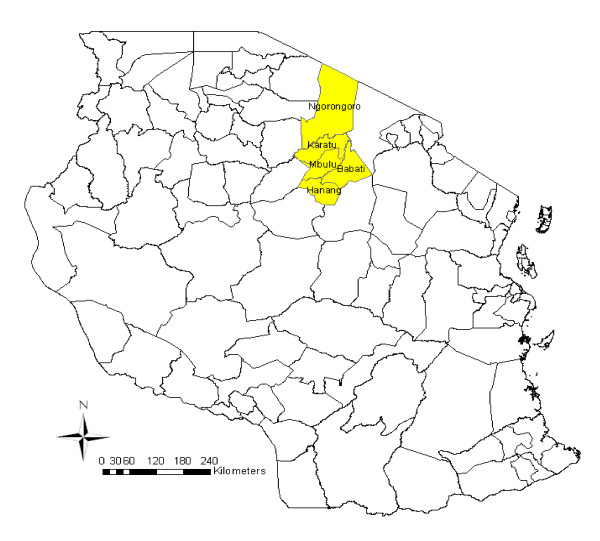
The map of Tanzania showing the study area.

The study was conducted in the northern regions of Arusha and Manyara, Tanzania. Districts involved with the study included Mbulu, Babati and Hanang in Manyara region and Ngorongoro and Karatu in Arusha region (Figure 1). Hospitals involved with the study included Babati and Dareda hospitals in Babati district, Mbulu and Hydom hospitals in Mbulu district, Katesh hospital in Hanang district, Karatu Lutheran hospital in Karatu district and Endulen and Wasso hospitals in Ngorongoro district.

### Study design, laboratory investigation and cases enrolment

This was designed as a longitudinal study. Any patient who presented to the hospitals between June 2002 and April 2003 with any of the clinical features including fever, headache, joint pain, malaise, backache, fatigue and loss of appetite was enrolled into the study by a practitioner attending the patient as a suspected brucellosis case. Other information such as personal particulars, data on clinical history, level of education, whether they keep livestock, economic status etc. were also recorded by an attending practitioner. Laboratory investigations at the health facilities using the rapid agglutination tests including the RBPT were conducted and patients were managed according to the clinical features and laboratory results. An aliquot of each sample was stored for c-ELISA test at the VLA in the UK. Any patient with any two or more of the clinical features such as fever, headache, joint pain, waist pain, backache, malaise, fatigue and tested positive with the c-ELISA test at the VLA was defined as a brucellosis case.

### Distance to the nearest hospital

The GIS coordinates of the households and of the hospitals were recorded from a hand-held Garmin^® ^GPS machine. All coordinates were then transferred to Excel 2003 spreadsheets (Microsoft Corporation, Redmond, Washington, USA) and the distance between the hospitals and the households calculated in kilometers using ArcGIS 9 software (ESRI, Redlands, California).

### Patient delay

Due to time constraints and difficulties in accessing brucellosis cases, the principal investigator was able to collect data on health-seeking behavior for 49 cases. Patient delay was defined as the time interval between the development of first symptoms of brucellosis to the time the case presented to hospital. Thirty days since the onset of first symptoms was taken as the cut-off point during which any patient with brucellosis symptoms was supposed to have presented to hospital for diagnosis and treatment [2]. All cases who presented to hospital 30 days or more after the onset of the first symptoms were defined as delayed going to hospital and those who presented earlier than 30 days not delayed.

### Data analysis

Data were entered on Excel 2003 spreadsheets (Microsoft Corporation, Redmond, Washington, USA) and analyzed using Minitab version 1.4 (Minitab Inc. 2000, Release 14 for Windows, State College, Pennsylvania). Chi-square tests were used to analyze all data and Fisher's exact test was used where 2 × 2 tabular results were obtained with any expected counts of less than 5. A p-value of < 0.05 was considered statistically significant. In multivariate analysis, Minitab version 1.4 was used to run logistic regression analysis. A backward stepwise method was used to find the best suite of variables (model simplification). The least significant variables were considered first for removal. Any variables that caused an insignificant increase in deviance on removal from the model was left out of the model while the variable that caused a significant increase in deviance on removal was retained in the model.

### Ethical issues

The study was peer reviewed and cleared for ethics by the Medical Research Coordinating Committee of the National Institute for Medical Research in the Republic of Tanzania. Verbal consent was also sought from all the patients before they were enrolled into the study and before any diagnostic procedure was conducted.

## Results

Of the 1586 samples that were collected from suspected brucellosis patients over a ten-month period and sent to VLA for the c-ELISA test, 98 (6.2%) tested positive for brucellosis. These 98 cases were considered to be confirmed positive cases.

### Patient delay and treatment delay

**Figure 2:**
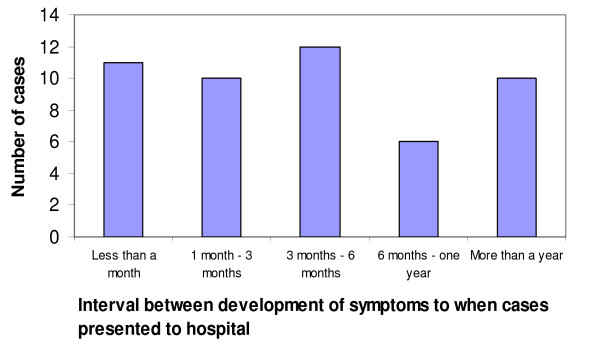
The time brucellosis cases presented to hospital after development of first symptoms.

Of the 49 cases whose data on health-seeking behavior was available, 11 (22.4%) went to hospital within one month after the onset of symptoms, 10 (20.4%) between one and three months, 12 (24.5%) between three and six months, six (12.2%) between six months and one year and 10 (20%) sought treatment more than a year after the onset of symptoms (Figure 2). Using the cut-off point of 30 days as the time that a case was supposed to have presented to hospital, the median patient delay time was 90 days (mean, 157. 3 days). Health system delay was a result of false negative results causing a failure to diagnose 22 (44.8%) cases of brucellosis on their first visits to hospitals.

### Site of first treatment

The majority of brucellosis cases 43 (87.7%) gave a history of going to hospital as the first point of care, five (10.2%) purchased drugs from a nearby drug shop before going to hospital and one patient (2%) went to a local traditional healer first

### Factors responsible for patient delays

The mean distance between the households of cases and hospitals was determined as 8.3 km, (median, 7.1 km). Univariate analysis showed that age of the case, distance to hospital, economic status, whether the household keeps livestock and knowledge of brucellosis were significantly associated with patient delay in presentation to hospitals (Table 1). In the multivariate analysis, patient delay was found to be most associated with distance to the nearest hospital, knowledge of brucellosis and if the household of a case keeps livestock (likelihood ratio p-value 0.03) (Table 2).

**Table 1:** Univariate analysis of factors responsible for patient delay

				**95% confidence interval**		
					
**Variable**	**Coefficients**	**Std. Error**	**Odds ratio**	**Lower**	**Upper**	**Likelihood ratio p-value**
Keeps livestock	1.61	0.63	5	1.45	17.27	0.003†
Distance to village centre	0.13	0.11	1.14	0.92	1.41	0.20
Religion	0.41	0.91	1.50	0.25	8.98	0.65
Tribe	0.69	0.71	2	0.50	7.99	0.14
	1.25	0.80	3.50	0.73	16.85	
Distance to hospital	0.11	0.06	3.12	1.00	4.26	0.02†
Gender	0.69	0.43	2	0.86	4.67	0.09
Age	0.02	0.01	1.02	1.01	1.04	0.001†
Knowledge of brucellosis	1.50	0.78	4.50	0.97	20.83	0.03†
Level of education	1.25	0.80	3.50	0.73	16.85	0.09
Economic status	1.47	0.64	4.33	1.23	15.21	0.02†
	0.69	0.87	2	0.37	10.92	
If any member of the household suffered from brucellosis	1.09	0.82	3	0.61	14.86	0.15

† – Variables included in multivariate analysis

**Table 2:** Final model of multivariate analysis of causes of patient delay

**Variable**	**Coefficients**	**Std. error**	**P-value**	**Odds ratio**	**95% confidence interval**	
					
					**Lower**	**Upper**
Distance to hospital	0.03	0.07	0.00	2.23	0.89	3.18
If keeps livestock	1.23	1.11	0.04	3.42	0.39	30.02
Knowledge of brucellosis	0.28	1.09	0.02	1.32	0.15	11.34

## Discussion

Cases that live far from hospitals were found to have a higher chance of delaying going to hospital compared to those living close to hospitals. In the study area, many households are far from health facilities and the infrastructures are still poor. Delay to present to hospital is therefore associated with how accessible the hospitals are. The government should try with little resources available to bring health care closer to the people particularly those in rural areas who make the majority of the population [30] and who form the workforce of the country.

Cases keeping livestock were also found to delay going to hospital. Possibly it was difficult for them to leave their livestock with nobody to attend to or they were too occupied with caring for the livestock causing them to have hardly any time to present to hospital. They probably presented to hospital when they were less engaged with caring for livestock. Since the majority of the people living in the study area keep livestock [31], awareness on the importance of going to hospital early requires considerable resources and commitment.

Cases with the knowledge of how brucellosis is transmitted and how it manifests also delayed going to hospital. Brucellosis is known to run a chronic course with some of the symptoms that may not be very severe at the onset and a fatality rate of 0.2% if untreated [32-34]. This could explain the reason why cases with the knowledge of brucellosis delayed going to hospital.

In the present study, the majority of brucellosis cases presented to hospital with a long history of symptoms. Some of the cases had been to hospital several times and had received treatment for other diseases such as malaria before being diagnosed as suffering from brucellosis. It became apparent that persistence of symptoms rather than the severity of the symptoms was the main complaint of brucellosis cases.

As a result of false negative results, 22 (44.8%) brucellosis cases were not diagnosed at the hospitals on their first presentations. These were treated for other diseases such as malaria which is much more common in the study area than brucellosis and hence they continued to suffer from brucellosis. The delay to present to hospitals could have caused brucellosis to turn into chronic form which could not be detected by the agglutinations tests performed at the hospitals [19].

The majority of the cases (87.8%) first sought treatment at a hospital and a few either treated themselves at home by buying medicine from a nearby drug shop (10.2%) or attended to a local traditional healer (2%). In the study area, a purely private health system has not been fully established, the majority of patients therefore, go to public hospitals which are run by government or religious groups (missionary hospitals). It was hence unlikely for cases to be delayed in private commercial health providers such as those found in urban areas. Studies conducted elsewhere on the causes of patients suffering form different to delay to present to hospital established that patients spent longer periods seeking treatment from traditional healers, profit making or private hospitals or delayed due to poor referral systems. This caused their financial resources to be wasted at times without getting proper treatment [24,35,36].

## Conclusion

More efforts need to be put on improving the accessibility of health facilities to the rural poor people who succumb to most of the diseases including zoonoses. Health education to the public should also include emphasis on patients to present to hospital early when chronic clinical features and complications have not developed as late presentation may carry poor prognosis even after treatment.

## Competing interests

The author(s) declare that they have no competing interests.

## Authors' contributions

SC, JF, NPF, RK, AM, KJ, and GS were involved in the design of the study. KJ, SC, JF, RK and GS supervised the fieldwork. SC, KJ, NPF, and MB assisted in data analysis and write up. SC, JF, NPF, RK, AM, KJ, DK and GS assisted in manuscript write-up.

All authors have read and approved the final manuscript

## Pre-publication history

The pre-publication history for this paper can be accessed here:



## References

[B1] Young EJ, Mandell GL, Bennet JE, Dolin R (2000). *Brucella *species. Principles and practice of infectious diseases.

[B2] Colmenero JD, Reguera JM, Martos F, Sánchez-De-Mora D, Delgado M, Causse M, Martin-Farfán A, Juárez C (1996). Complications associated with *Brucella melitensis *infection: a study of 530 cases. Medicine (Baltimore).

[B3] McDermott JJ, Arimi SM (2002). Brucellosis in sub-Saharan Africa: epidemiology, control and impact. Veterinary Microbiology.

[B4] Smits HL, Cutler SJ (2004). Contributions of biotechnology to the control and prevention of brucelliosis in Africa. African Journal of Biotechnology.

[B5] Mutanda LN (1998). Selected laboratory tests in febrile patients in Kampala, Uganda. East African Medical Journal.

[B6] Maichomo MW, McDermott JJ, Arimi SM, Gathura PB (1998). Assessment of the Rose-Bengal plate test for the diagnosis of human brucellosis in health facilities in Narok district, Kenya. East African Medical Journal.

[B7] Maichomo MW, McDermott JJ, Arimi SM, Gathura PB, Mugambi TJ, Muriuki SM (2000). Study of brucellosis in pastoral community and the evaluation of the usefulness of clinical signs and symptoms in differentiating it from other flu-like diseases. African Journal of Health Sciences.

[B8] FAO/WHO. (1985) (1986). Joint Expert Committee on Brucellosis. Meeting held in Geneva from 12 to 19 November.

[B9] Gotuzzo E, Carrillo C, Guerra J, Llosa L (1986). An evaluation of diagnostic methods for brucellosis the value of bone marrow culture. Journal of Infectious Diseases.

[B10] Araj GF, Lulu AR, Khateeb MI, Haj M (1990). Specific IgE response in patients with brucellosis. Epidemiology and Infection.

[B11] Corbel MJ, Brinley-Morgan WJ, Krieg NR, Holt JG (1984). Genus *Brucella*. Bergey's manual of systematic bacteriology.

[B12] Alton GG, Corner LA, Plackett P (1983). The role of the differential complement fixation test, using rough and smooth *Brucella *antigens, in the anamnestic test. Australian Veterinary Journal.

[B13] Nielsen K, Lin M, Gall D, Jolley M (2000). Fluorescence polarization immunoassay: detection of antibody to *Brucella abortus*. Methods.

[B14] Lucero NE, Escobar GI, Ayala SM, Silva PP, Nielsen K (2003). Fluorescence polarization assay for diagnosis of human brucellosis. Journal of Medical Microbiology.

[B15] Lucero NE, Foglia L, Ayala SM, Gall D, Nielsen K (1999). Competitive enzyme immunoassay for diagnosis of human brucellosis. Journal of Clinical Microbiology.

[B16] Parratt D, Nielsen KH, White RG (1977). Radioimmunoassay of IgM, IgG, and IgA *Brucella *antibodies. Lancet.

[B17] Colmenero JD, Reguera JM, Cabrera FP, Hernandez S, Porras J, Manchado P, Miranda MT (1989). [Combined use of Rose Bengal and indirect immunofluorescence in the diagnosis of brucellosis]. Enfermedades Infecciosas Microbiología Clínica.

[B18] Reddin JL, Anderson RK, Jenness JR, Spink WW (1965). Significance of 7S and macrogloblulin *Brucella *agglutinins in human brucellosis. The New England Journal of Medicine.

[B19] Mittal KR, Tizard IR (1983). Agglutination tests and their modifications in the diagnosis of bovine brucellosis. Comparative Immunology, Microbiology and Infectious Diseases.

[B20] Muriuki SM, McDermott JJ, Arimi SM, Mugambi JT, Wamola IA (1997). Criteria for better detection of brucellosis in the Narok District of Kenya. East African Medical Journal.

[B21] Oomen LJ, Waghela S (1974). The Rose Bengal plate test in human brucellosis. Tropical and geographical medicine.

[B22] Corbel MJ (1997). Brucellosis: An overview. Emerging Infectious Diseases.

[B23] Domenech J, Corbel MJ, Thomas EL, Lucet P (1983). [Bovine brucellosis in central Africa: VI. Identification and typing of isolated strains in Chad and Cameroon]. Revue D'élevage et de Médecine vétérinaire des Pays Tropicaux.

[B24] Wandwalo ER, Morkve O (2000). Delay in tuberculosis case-finding and treatment in Mwanza, Tanzania. The International Journal of Tuberculosis and Lung Disease.

[B25] Yimer S, Bjune G, Alene G (2005). Diagnostic and treatment delay among pulmonary tuberculosis patients in Ethiopia: A cross sectional study. BMC Infectious Diseases.

[B26] Odusanya OO, Babafemi JO (2004). Patterns of delays amongst pulmonary tuberculosis patients in Lagos, Nigeria. BMC Public Health.

[B27] Kiwuwa MS, Charles K, Harriet MK (2005). Patient and health service delay in pulmonary tuberculosis patients attending a referral hospital: a cross-sectional study. BMC Public Health.

[B28] Odiit M, Shaw A, Welburn SC, Fevre EM, Coleman PG, McDermott JJ (2004). Assessing the patterns of health-seeking behaviour and awareness among sleeping-sickness patients in eastern Uganda. Annals of Tropical Medicine and Parasitology.

[B29] Mwende J, Bronsard A, Mosha M, Bowman R, Geneau R, Courtright P (2005). Delay in presentation to hospital for surgery for congenital and developmental cataract in Tanzania. The British Journal of Ophthalmology.

[B30] Tanzania National population and housing census results of 2002. http://www.tanzania.go.tz/census/.

[B31] Ministry of Agriculture and Cooperatives. Agriculture census report 1994/95, The United Republic of Tanzania.

[B32] Hurvell B, Ahvonen P, Thal E (1971). Serological cross-reactions between different *Brucella species *and *Yersinia enterocolitica*. Agglutination and complement fixation. Acta Veterinaria Scandinavica.

[B33] Jacobs F, Abramowicz D, Vereerstraeten P, Le Clerc JL, Zech F, Thys JP (1990). *Brucella *endocarditis: the role of combined medical and surgical treatment. Reviews of Infectious Diseases.

[B34] Young EJ (1995). Brucellosis: current epidemiology, diagnosis, and management. Current Clinical Topics in Infectious Diseases.

[B35] Anorlu RI, Orakwue CO, Oyeneyin L, Abudu OO (2004). Late presentation of patients with cervical cancer to a tertiary hospital in Lagos: what is responsible?. Eur J Gynaecol Oncol.

[B36] Anonymous (1984). Survey of the previous investigation and treatment by private practitioners of patients with pulmonary tuberculosis attending government chest clinics in Hong Kong.

